# Risk Assessment and Risk-Adapted Treatment Selection: A Case-Based Approach for Chronic Lymphocytic Leukemia

**Published:** 2017-07-01

**Authors:** Sandra Kurtin, Ali McBride

**Affiliations:** University of Arizona and Arizona Cancer Center, Tucson, Arizona

## Abstract

Selected Patient Cases From the APSHO Regional Lecture Series

**INTRODUCTION**

As the official publication of the Advanced Practitioner Society for Hematology and Oncology (APSHO), JADPRO is pleased to offer Part 3 of an accredited educational activity based on the recently concluded APSHO Regional Lecture Series. Hosted in collaboration with major cancer centers around the country, the APSHO Regional Lecture Series brought case-based didactic presentations and skills workshops to advanced practitioners.

In the spirit of JADPRO, three accredited Grand Rounds articles by Beth Eaby-Sandy, MSN, CRNP, OCN® (non–small cell lung cancer) and Sandra Kurtin, PhDc, ANP-C, AOCN® (multiple myeloma and chronic lymphocytic leukemia)—program chairs for the regional lecture series—offer the same practice-changing information and strategies for advanced practitioners.

In this Grand Rounds article, program chair Sandra Kurtin reviews the prognosis, risk assessment, and treatment for patients with chronic lymphocytic leukemia and assesses four case studies.

You can read Parts 1 and 2 in previous issues of JADPRO or online at advancedpractitioner.com. Check out apsho.org/lectures for information on registering for upcoming JADPRO Regional Lectures this year at a location near you.

**Risk Assessment and Risk-Adapted Treatment Selection: A Case-Based Approach for Chronic Lymphocytic Leukemia**

This activity is supported by an educational grant from Pharmacyclics, LLC, an AbbVie Company, and Janssen Biotech, Inc.

A continuing education article for nurse practitioners, clinical nurse specialists, advanced degree nurses, and oncology and hematology nurses.

**Release date:** July 18, 2017

**Expiration date:** November 13, 2017

**Expected time to complete activity:** 0.5 hour

© 2017, Meniscus Educational Institute. All rights reserved.

**Meniscus Educational Institute**

3131 Princeton Pike

Building 1, Suite 205A

Lawrenceville, NJ 08648

Voice: 609-246-5000

Fax: 609-449-7969

E-mail: lrubin@harborsidemeded.com

**Journal of the Advanced Practitioner in Oncology**

94 N. Woodhull Road

Huntington, NY 11743

Voice: 631-692-0800

Fax: 631-692-0805

E-mail: claudine@harborsidepress.com

© *2017, Meniscus Educational Institute. All rights reserved.*

**Faculty**

**Sandra Kurtin, PhDc, ANP-C, AOCN®**, University of Arizona and Arizona Cancer Center, Tucson, Arizona

**Ali McBride, PharmD, BCOP**, University of Arizona and Arizona Cancer Center, Tucson, Arizona

## Activity Rationale and Purpose

The therapeutic landscape of chronic lymphocytic leukemia (CLL) continues to change rapidly. Advances in pathology and genomics are also enabling researchers to use genetic tests to make more accurate risk assessments for various subgroups of CLL patients. Translating these advances into improved quality of care at the bedside poses a challenge; meeting this challenge will require continued education for advanced practitioners who are increasingly important members of the oncology care team.

## Intended Audience

The activity’s target audience will consist of nurse practitioners, clinical nurse specialists, advanced degree nurses, and oncology and hematology nurses.

## Learning Objectives

After completing this educational activity, participants should be able to:

- Apply the principles of risk-adapted treatment using case-based scenarios to illustrate the impact of patient attributes and disease specific attributes
- Manage toxicities associated with newer agents used to treat chronic lymphocytic leukemia
- Describe the role of oral therapies in treating patients with chronic lymphocytic leukemia

## Continuing Education

Statement of Credit—Participants who successfully complete this activity (including the submission of the post-test and evaluation form) will receive a statement of credit.

**Nurses**. This activity for 0.5 contact hour is provided by the Meniscus Educational Institute.

The Meniscus Educational Institute is accredited as a provider of continuing nursing education by the American Nurses Credentialing Center’s Commission on Accreditation. 

Provider approved by the California Board of Registered Nursing, Provider No. 13164, for 0.5 contact hour.

## Financial Disclosures

All individuals in positions to control the content of this program (eg, planners, faculty, content reviewers) are expected to disclose all financial relationships with commercial interests that may have a direct bearing on the subject matter of this continuing education activity. Meniscus Educational Institute has identified and resolved all conflicts of interest in accordance with the MEI policies and procedures. Participants have the responsibility to assess the impact (if any) of the disclosed information on the educational value of the activity. 

**Faculty**

**Sandra Kurtin, PhDc, ANP-C, AOCN®**, has served as a consultant for AbbVie, Celgene, and Genentech.

**Ali McBride, PharmD, BCOP**, has served on the speakers bureau for AbbVie.

**Lead Nurse Planner**

**Dorothy Caputo, MA, BSN, RN**, has nothing to disclose.

**Nurse Planner**

**Wendy J. Smith, ACNP, AOCN®**, has nothing to disclose.

**Planners**

**Jeannine Coronna** has nothing to disclose.

**Claudine Kiffer** has nothing to disclose.

**Pamela Hallquist Viale, RN, MS, CNS, ANP**, has nothing to disclose.

**Patti McLafferty** has nothing to disclose.

**Lynn Rubin** has nothing to disclose.

**Annie Yueh** has nothing to disclose.

**Content Reviewers**

**Leslie Lauersdorf, ARNP, AOCNP**, has served on the speakers bureau for Amgen.

## Disclaimer

This activity has been designed to provide continuing education that is focused on specific objectives. In selecting educational activities, clinicians should pay special attention to the relevance of those objectives and the application to their particular needs. The intent of all Meniscus Educational Institute educational opportunities is to provide learning that will improve patient care. Clinicians are encouraged to reflect on this activity and its applicability to their own patient population.

The opinions expressed in this activity are those of the faculty and reviewers and do not represent an endorsement by Meniscus Educational Institute of any specific therapeutics or approaches to diagnosis or patient management.

## Product Disclosure

This educational activity may contain discussion of published as well as investigational uses of agents that are not approved by the US Food and Drug Administration. For additional information about approved uses, including approved indications, contraindications, and warnings, please refer to the prescribing information for each product.

## How to Earn Credit

To access the learning assessment and evaluation form online, visit http://meded.hbrsd.com/

**Statement of Credit:** Participants who successfully complete this activity (including scoring of a minimum of 70% on the learning assessment and complete and submit the evaluation form with an E-mail address) will be able to download a statement of credit.

## Risk Assessment and Risk-Adapted Treatment Selection: A Case-Based Approach for Chronic Lymphocytic Leukemia

Chronic lymphocytic leukemia (CLL) is the most common form of leukemia in the United States, with roughly 19,000 new cases diagnosed in 2016 (Siegel, Miller, & Jemal, 2017). The median age at diagnosis is 71 years, with nearly 70% of newly diagnosed patients over the age of 65 (Siegel et al., 2017; Stauder et al., 2016). Chronic lymphocytic leukemia is incurable in the absence of an allogeneic stem cell transplant. The disease trajectory is characterized by periods of stable disease, relapse, and in some cases Richter’s transformation. The 5-year relative survival rates have increased from 67.5% (1975–1977) to 81.7% (2005–2011); however, the clinical course of CLL is highly variable, ranging from indolent disease with a median survival exceeding 15 years without treatment, to more aggressive and relatively treatment-resistant disease with median survival of less than 4 years (Eichhorst & Hallek, 2016). This variability in life expectancy is in large part based on the molecular underpinnings of the disease, individual patient characteristics, and access to care.

**Figure 1:**
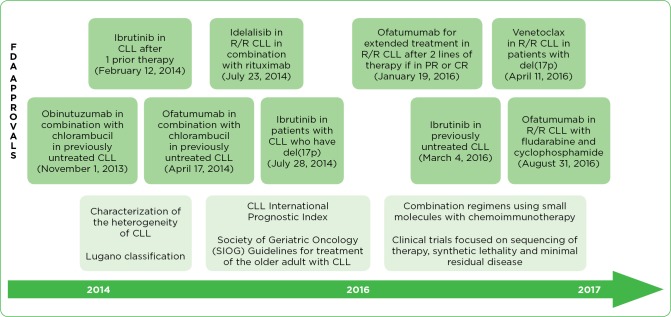
Scientific and therapeutic innovation in chronic lymphocytic leukemia 2013–2017. R/R = relapsed/refractory; PR = partial response; CR = complete response. Information from Balducci & Dolan (2015); Parikh & Shanafelt (2016); Pflug et al. (2014); Rai & Jain (2016); Wierda (2015); Wierda et al. (2017).

There have been significant changes in the therapeutic landscape for CLL, due to enhanced understanding of the molecular features of CLL (Figure 1). In addition, several consensus statements and updated clinical guidelines have been developed. Personalized, tailored therapies based on risk assessment and risk-adapted treatment selection are now the standard of care. Chemoimmunotherapy and small-molecule agents have offered expanded options for treatment, including first-in-class agents. There have been nine US Food and Drug Administration (FDA) approvals for new drugs or expanded applications for existing compounds in less than 3 years. The research pipeline is robust, with additional approvals anticipated in the next 1 to 2 years. These advances offer hope to patients with CLL but require careful application of both diagnostic and prognostic strategies to achieve the improved clinical outcomes demonstrated in clinical trials.

The implications for oncology providers, including the advanced practitioner, are multifaceted. Lifelong learning, involvement in scientific discovery, vigilance in observing and reporting clinical experiences with novel agents in the postmarketing setting, familiarity with metrics for value-based care, and a commitment to improving patient outcomes will be necessary. The purpose of this article is to provide the advanced practitioner in oncology with an update on risk assessment and risk-adapted treatment selection in patients with CLL using a case-based approach to demonstrate application of emerging data. The focus will be on the management of patients with currently approved agents, with mention of emerging therapies.

## PREDICTIVE AND PROGNOSTIC FEATURES OF CHRONIC LYMPHOCYTIC LEUKEMIA

Chronic lymphocytic leukemia is characterized by mature B lymphocytes with an immunophenotype including CD5+, CD19+, CD20 weak, CD23+, and weak-surface expression of monoclonal immunoglobulin (SmIg-weak; Gruber & Wu, 2014). Small lymphocytic lymphoma (SLL) has the same immunophenotype as CLL but includes a predominance of nodal disease, bone marrow involvement, organomegaly, and < 5,000 tumor cells/μL. The clinical characteristics of the disease provided the basis for staging and prognosis for the past 50 years.

More recently, advances in flow cytometry, fluorescence in situ hybridization (FISH), and next-generation sequencing (NGS) have elucidated features of the disease that confer either a favorable or unfavorable clinical course—and in some cases implications for treatment (Table 1). Features considered to confer an inferior survival advantage and in some cases refractory disease include the deletion of 17p or mutation of *TP53*, the presence of 11q or a complex karyotype (> 3 abnormalities in > 1 cell), < 2% (unmutated) immunoglobulin heavy-chain gene variable region (IgHV) mutation (uIgHV), > 30% CD38, and > 20% zeta-chain-associated protein 70 (ZAP-70; Parikh & Shanafelt, 2016). 

Patients with uIgHV tend to have more aggressive disease, high expression of CD38, ZAP-70–positive disease, poor risk genetic profiles, and shorter survival (Montserrat et al., 2016). In a recent systematic review and meta-analysis including 31 studies, uIgHV was associated with inferior median progression-free survival (PFS; range, 1–5 years) and median overall survival (OS; range, 3.2–10 years) compared with patients with mutated IgHV (median PFS range, 9.2–18.9 years; median OS range, 17.9–25.8 years; Parikh, Strati, Tsang, West, & Shanafelt, 2016). In this same analysis, patients with del(17p13) and del(11q23), considered FISH high risk, had significantly shorter median PFS (range, 0.1–5.2 years) and median OS (range, 3.3–9.7 years) compared with patients with FISH low/intermediate-risk profiles including del(13q), normal, and trisomy 12 (median PFS range, 1.5–22 years; median OS range, 7.5–20.5 years; Parikh et al., 2016). Chronic lymphocytic leukemia with high expression of CD38 confers a less favorable prognosis due to the association of CD38 with migration and homing of CLL cells to secondary lymphoid organs. The malignant cells receive support from the tumor microenvironment in these lymphoid organs, causing suppression of immune surveillance through the production of tolerogenic compounds and increasing the risk of Richter’s transformation (Burgler, 2015; Parikh & Shanafelt, 2014). The tyrosine kinase ZAP-70, involved in cellular signaling of T cells, has been shown to correlate with the presence of uIgHV. 

The heterogeneity of CLL is compounded by clonal evolution characterized by acquired genetic and epigenetic changes, which give rise to new subclones with emergence of one or more fitter or resistant subclones that become dominant (Guièze & Wu, 2015). Therefore, full characterization of the disease at the time of initial diagnosis and at disease progression is critical to effective risk analysis, prognostication, and clinical management of CLL. This includes reimaging; excisional biopsies in the case of rapidly progressive adenopathy; and a bone marrow biopsy with flow cytometry, FISH, and NGS when available to obtain a full molecular profile (Eichhorst & Hallek, 2016). Reevaluation of comorbidities and fitness for treatment is necessary for the optimal selection of treatment.

## RISK ASSESSMENT AND PROGNOSTICATION

Given the heterogeneity of the disease, an international consortium of CLL experts convened to develop an internationally applicable prognostic index for CLL: the CLL-International Prognostic Index (CLL-IPI; 2016). Based on a retrospective analysis of 26 different items, 5 items were found to be independent predictors for OS (age, clinical stage, 17p del and/or *TP53* mutation, IgHV status, and β₂ microglobulin). Each item was weighted according to regression analysis, with the presence of del(17p) or *TP53* mutation carrying the highest weight (score of 4; hazard ratio [HR]: 4.2; *p*< .001). When combined, these five factors produced four groups with significantly different OS rates (93%, 79%, 64%, and 23% at 5 years; *p*< .001; the International CLL-IPI Working Group, 2016; Table 2). 

**Table 1:**
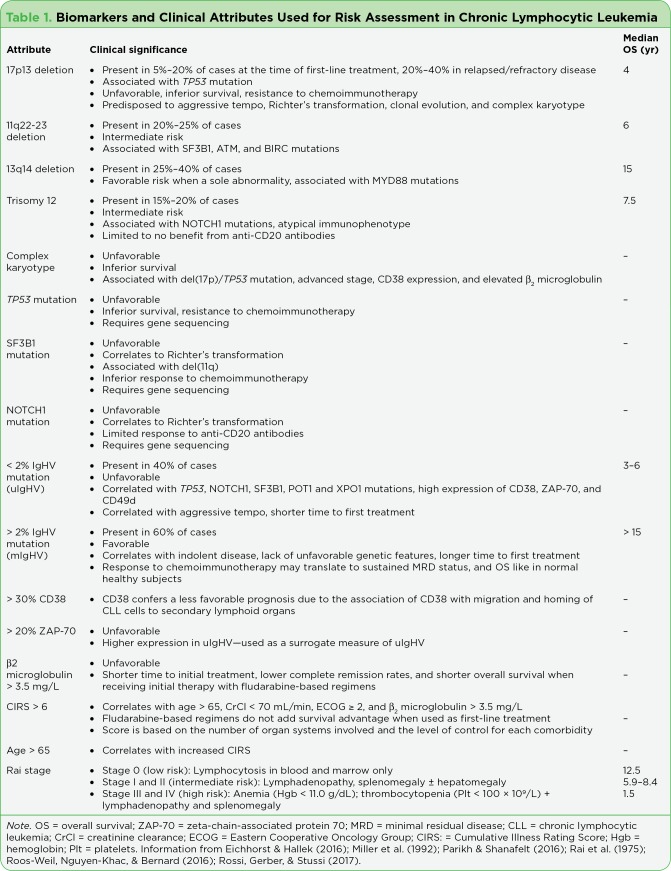
Biomarkers and Clinical Attributes Used for Risk Assessment in Chronic Lymphocytic Leukemia

Given the older age of most patients with CLL, consideration of fitness and frailty as well as the presence of comorbidities is crucial when considering treatment (Goede et al., 2014; Stauder et al., 2016). The presence of comorbidities, particularly when poorly controlled, is associated with an increased risk of treatment-related adverse events, inferior survival, decreased access to care, and inferior quality of life (Sarfati et al., 2016).

## RISK-ADAPTED TREATMENT SELECTION

**Figure 2:**
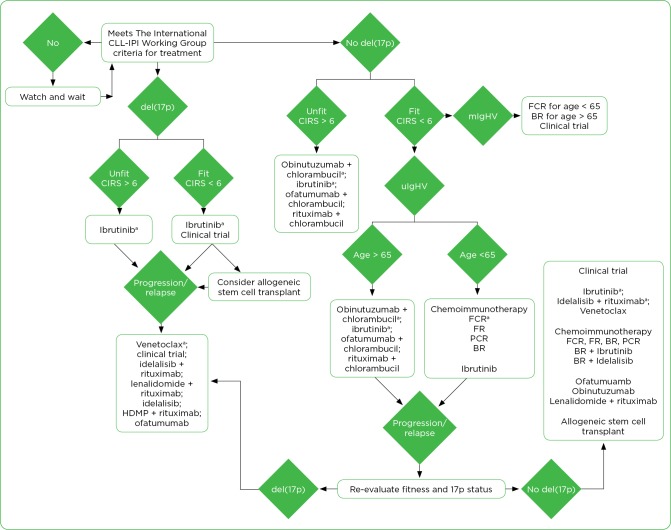
Risk-adapted treatment of chronic lymphocytic leukemia. CIRS = Cumulative Illness Rating Scale; HDMP = high-dose methylprednisolone; ulgHV = unmutated immunoglobulin heavy-chain variable gene; mlgHV = mutated IgHV; FCR = fludarabine, cyclophosphamide, and rituximab; FR = fludarabine and rituximab; PCR = pentostatin, cyclophosphamide, rityximab; BR = bendamustine and rituximab. ^a^NCCN Category 1 designation.

The goals of therapy in CLL include early identification of high-risk disease, estimation of the time to treatment, early identification of lack of or loss of response to treatment, prevention of infections, preservation of future treatment options by limiting end-organ damage and cumulative treatment toxicity, and ultimately maintenance or improvement in quality of life. Applying the findings from the risk assessment will help to avoid overtreatment and unnecessary exposure to adverse events as well as undertreatment and suboptimal therapeutic benefit. Risk-adapted treatment, including consideration of disease attributes, line of therapy, and patient attributes, is essential to maximizing outcomes and quality of life (Figure 2). All patients should receive palliative and supportive care, which should be tailored to the individual patient and regimen and initiated at the time of diagnosis. Potential disease and treatment-emergent adverse events must be considered in the context of individual patient attributes when considering the risks associated with treatment (Table 3). 

**Table 2:**
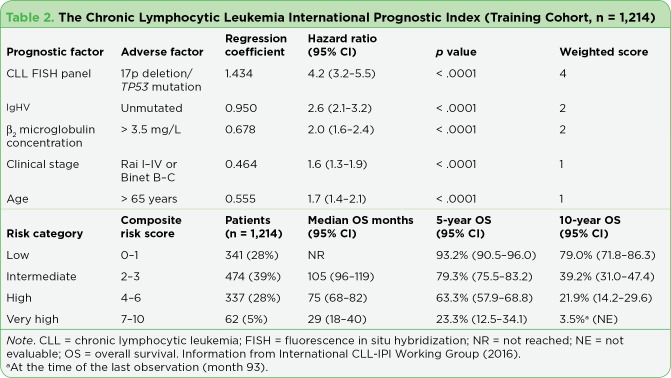
The Chronic Lymphocytic Leukemia International Prognostic Index (Training Cohort, n = 1,214)

## TREATMENT OF NEWLY DIAGNOSED CHRONIC LYMPHOCYTIC LEUKEMIA

It is tempting to initiate treatment early in patients with CLL, given the number of newly developed agents with unique mechanisms of action and promising outcomes. However, there are no data to date to change the recommendations for treatment set forth by the International CLL-IPI Working Group (Table 2). 

Let’s apply the metrics of risk assessment to three patients with previously untreated CLL.

**Case Study 1**

Mrs. J is a 75-year-old female who presented to her primary care physician (PCP) 2 weeks ago with palpitations, progressive fatigue, and increased joint pain. She was noted to have an elevated white blood cell (WBC) count (96 × 1,000/μL), moderate anemia (hemoglobin [Hgb] 8.1 g/dL), and thrombocytopenia (97 × 1,000/μL). Flow cytometry on the peripheral blood confirms a diagnosis of CLL. A bone marrow biopsy is performed showing 90% involvement of the bone marrow by CLL, normal female karyotype, normal FISH panel, ZAP-70–negative, β₂ microglobulin 3.4 m/L, and mutated IgHV. A noncontrast computed tomography (CT) shows no significant adenopathy and no splenomegaly. She has a history of atrial fibrillation, chronic renal insufficiency (creatinine clearance [CrCl] 55 mL/min), and osteoarthritis. She is married and lives with her husband, who is relatively healthy. They walk with their dog daily. 

 *Risk Assessment:* 

- Rai stage: III
- Cumulative Illness Rating Scale (CIRS) = 3
- CLL-IPI score: 2 (age > 65, Rai stage III) = intermediate risk
- The International CLL-IPI Working Group criteria for treatment: Yes—progressive fatigue, cytopenias, fevers without infection
- Risk-adapted treatment selection: either a clinical trial or the combination of bendamustine plus rituximab

**Case Study 2**

Mrs. R is a 72-year-old female who presented with recurring pharyngitis, low-grade fevers, progressive fatigue, adenopathy, abdominal pain, and > 10% weight loss. Her medical history includes hypertension, gastroesophageal reflux disease, hypothyroidism, cholelithiasis, shingles, exaggerated reaction to insect bites, and chronic pain due to a previous car accident. She was diagnosed with CLL 1 year ago based on peripheral blood analysis and has been monitored off treatment.

On a physical exam, she has extensive adenopathy in the cervical, axillary, and inguinal regions as well as palpable splenomegaly. Other diagnostic findings include: WBC count: 230.7 × 1,000/∝L; absolute lymphocyte count (ALC): 223.78 × 1,000/∝L; Hgb: 10.9 g/dL; platelets: 180 × 1,000/∝L; lactate dehydrogenase (LDH): 250 IU/L (upper limit of normal [ULN] 243), β₂ microglobulin : 2.5 mg/L, ZAP-70–positive. A bone marrow biopsy is performed, showing 80% involvement with small mature noncleaved lymphocytes, metaphase cytogenetics with a normal female karyotype (46, XX [15]), and FISH with 17p13 deletion in 92/200 cells. 

 *Risk Assessment:* 

- Rai stage: III (Hgb < 11 g/dL, splenomegaly, adenopathy, lymphocytosis)
- CIRS: 5
- CLL-IPI score: 8 (17p+, Rai stage I–IV, IgHV unmutated, age > 65) = very high risk
- The International CLL-IPI Working Group criteria for treatment: Yes—progressive fatigue, cytopenias, fevers without infection
- Risk-adapted treatment selection: either a clinical trial or ibrutinib (Imbruvica)

**Case Study 3**

Mr. B. is a 75-year-old male diagnosed as Binet stage 0 CLL. His disease profile includes small to medium adenopathy (0.7 cm–2.7 cm), FISH with trisomy 11q, mutated IgHV (mIgHV). His medical history includes psoriasis, seasonal sinus infections, and benign prostatic hyperplasia (BPH). He is here for a scheduled follow-up visit. Labs: WBC (25.5 × 1,000/∝L); ALC 18.62 × 1,000/∝L; normal β₂ microglobulin, and no cytopenias. He does report fatigue, but this has not changed. He is recently widowed and is depressed.

**Table 3:**
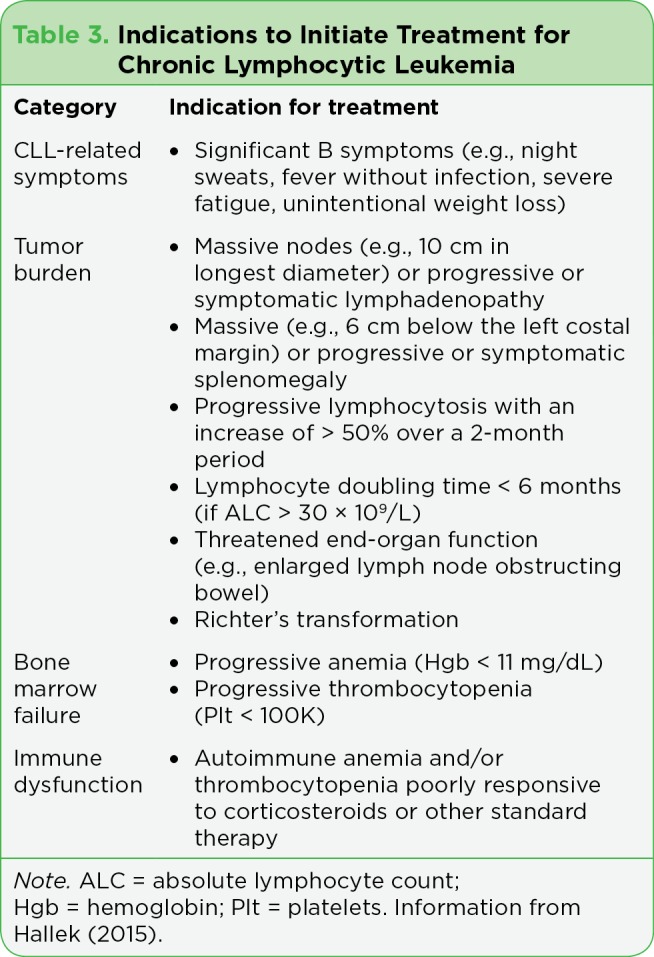
Indications to Initiate Treatment for Chronic Lymphocytic Leukemia

Recent publications evaluating long-term outcomes for CLL patients treated with ibrutinib in both TNCLL and RRCLL have elucidated several important points: (1) Uncontrolled adverse events continue to be a leading cause of treatment discontinuation–the most common adverse events cited for early discontinuation of ibrutinib included atrial fibrillation, infection, pneumonitis, bleeding, and diarrhea (Barr et al., 2016; Byrd et al., 2017; Jain et al., 2017; Mato, 2016); (2) Adverse-event profiles in the postmarketing setting are mostly consistent with profiles reported in the clinical trials, with some variations in frequency and severity in patients on long-term treatment including persistent hypertension and the incidence of atrial fibrillation and bleeding episodes (Barr et al., 2016; Gashonia et al., 2017; Kunk et al., 2016; Mato et al., 2016; Shanafelt et al., 2017; Yun, Vincelette, Acharya, & Abraham, 2017); (3) Patients who discontinued ibrutinib due to progression of disease may benefit from other small-molecule agents; however, those who progress with Richter’s transformation have an extremely poor prognosis, with an estimated life expectancy of 1.5 years (Mato et al., 2016). 

Pretreatment risk assessment is critical to mitigating risks and should include collaboration with co-managing providers such as PCPs and cardiologists to reduce potential drug-drug interactions and optimally manage expected adverse events. Factors shown to correlate with an increased risk of treatment-emergent atrial fibrillation include older age (≥ 75 years), male sex, valvular heart disease, and hypertension (Shanafelt et al., 2017; Yun et al., 2017). Anticoagulation, a proven method for reducing the potential for ischemic stroke in the setting of atrial fibrillation, can be safely managed in CLL patients receiving ibrutinib but requires vigilance and engagement of the patient in early reporting of adverse events and maintaining safety (Yun et al., 2017). Infections, particularly sinopulmonary infections, are common in the general CLL population. Atypical infections are also more common in CLL, owing to chronic immunosuppression associated with the disease and treatment. Vigilance in monitoring and methods to prevent atypical infections is recommended.

 *Risk Assessment:* 

- Rai stage: 0 (asymptomatic)
- CIRS: 1
- CLL-IPI score: 1 (age > 65) = low risk
- The International CLL-IPI Working Group criteria for treatment: No—he has no indications to treat
- Risk-adapted treatment selection: surveillance/watch and wait

**Discussion**

These case studies illustrate the significance of a comprehensive risk assessment to determine the need for treatment and the selection of therapy. The patient in case study 3 does not meet the International CLL-IPI Working Group criteria for treatment. Although he has the del(11q), a higher-risk feature, this alone does not indicate the need for treatment (International CLL-IPI Working Group, 2016). The patients in case study 1 and 2 meet the International CLL-IPI Working Group criteria to initiate treatment; however, the preferred regimen for treatment in each case is very different. 

**Table 4:**
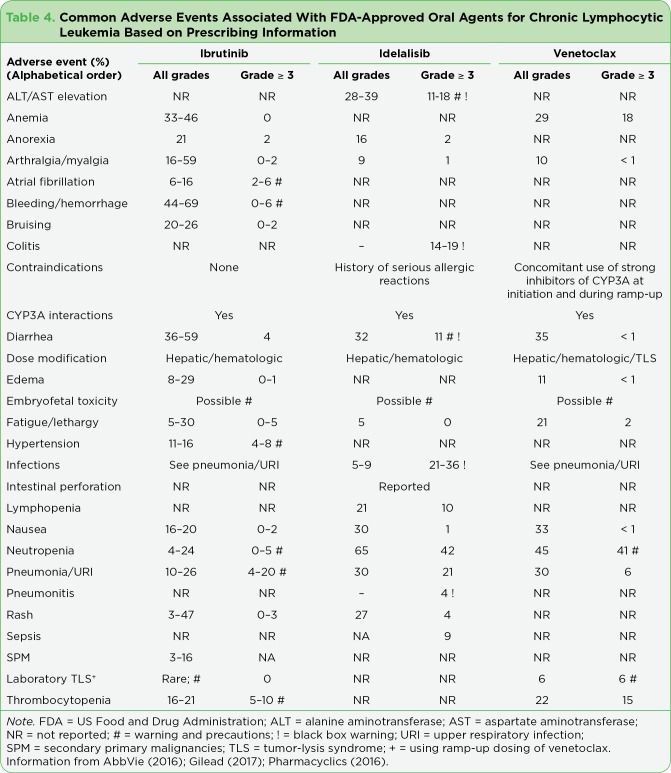
Common Adverse Events Associated with FDA-Approved Oral Agents for Chronic Lymphocytic Leukemia Based on Prescribing Information

In case study 1, the patient has mIgHV, with intermediate-risk disease. Patients with mIgHV in the absence of any other adverse risk factors may enjoy a longer time to treatment initiation. In addition, once treated with fludarabine, cyclophosphamide, and rituximab (FCR), these patients may experience long-term remissions and may in fact be potentially cured of their disease (Fischer et al., 2016; Thompson et al., 2016). This patient is older and does have comorbidities, which would increase the risk associated with FCR. Patients who are not candidates for this more intensive chemoimmunotherapy may benefit from bendamustine and rituximab (BR).

In the phase III CLL 10 trial comparing front-line BR and FCR in 561 patients with a CIRS < 6 and CrCl > 70 mL/min, without del(17p), median PFS was 41.7 months (95% confidence interval [CI] = 34.9–45.3) with bendamustine and rituximab and 55.2 months (95% CI not evaluable) with FCR (HR, 1.643, 90.4%; CI = 1.308–2.064) at 37 months of follow-up (Eichhorst et al., 2016). Objective response rate (ORR) was equivalent in each treatment group (95.4 vs. 95.7%;*p*= 1.0); however, patients in the FCR group were significantly more likely than those treated with BR to achieve a complete response (39.7 vs. 30.8%;*p*= .034). No differences in OS between the FCR and BR groups were noted at 36 months of follow-up (90.6 vs. 92.2%;*p*= .897). Adverse events, including severe neutropenia and infections, were more frequently observed with FCR when compared with BR, and serious infections were more common in patients over the age of 65 receiving FCR (Eichhorst et al., 2016). Therefore, FCR is not recommended for older or frail patients.

The patient in case study 2 has very high-risk disease, with a CLL-IPI score of 8. The presence of del(17p) accounts for 4 points alone. Standard chemoimmunotherapy is not effective in patients with del(17p). There are only two agents approved for treatment specifically in the presence of del(17p), including ibrutinib and venetoclax (Venclexta). Alemtuzumab has established activity in this setting but is no longer commercially available for treatment of CLL; however, it can be obtained for clinical use. It is not effective in patients with bulky disease and is associated with a higher risk of reactivation of cytomegaloviral infections (Wierda et al., 2017).

**Ibrutinib**

Ibrutinib, a first-in-class Bruton’s tyrosine kinase (BTK) inhibitor, has a broad indication in the treatment of CLL in treatment-naive (TN), relapsed or refractory (RR), and del(17p) CLL patients as well as those over the age of 65 (Foluso, Glick, Stender, & Jaiyesimi, 2016; Maddocks & Jones, 2016). It was first approved in February of 2014 as a single agent for treatment of patients with CLL after one prior therapy (Byrd et al., 2013, 2014). Continued clinical trials using ibrutinib as a single agent and in combination with BR led to its expanded approval for use in the front-line setting, including for older patients (Burger et al., 2015; O’Brien et al., 2014), and in combination with BR in patients with RRCLL (Chanan-Khan et al., 2016).

Among the 656 CLL patients participating in these trials, the ORR ranged from 63% to 88% in RRCLL and 97% in TN CLL, including patients with del(17p), 11q, and uIgHV (Farooqui et al., 2015; Foluso et al., 2016; Jain et al., 2017). Although response rates and PFS outcomes in these trials are promising, complete responses were rare, indicating a need to explore other novel combinations (Jain et al., 2017). Several trials are underway evaluating these novel combinations as well as second-generation BTK inhibitors (Table 5). 

**Table 5:**
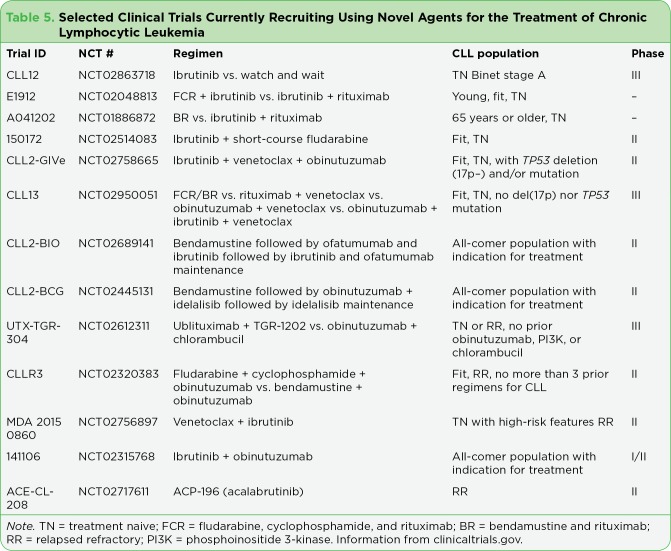
Selected Clinical Trials Currently Recruiting Using Novel Agents for the Treatment of Chronic Lymphocytic Leukemias

Recent publications evaluating long-term outcomes for CLL patients treated with ibrutinib in both TNCLL and RRCLL have elucidated several important points: (1) Uncontrolled adverse events continue to be a leading cause of treatment discontinuation–the most common adverse events cited for early discontinuation of ibrutinib included atrial fibrillation, infection, pneumonitis, bleeding, and diarrhea (Barr et al., 2016; Byrd et al., 2017; Jain et al., 2017; Mato, 2016); (2) Adverse-event profiles in the postmarketing setting are mostly consistent with profiles reported in the clinical trials, with some variations in frequency and severity in patients on long-term treatment including persistent hypertension and the incidence of atrial fibrillation and bleeding episodes (Barr et al., 2016; Gashonia et al., 2017; Kunk et al., 2016; Mato et al., 2016; Shanafelt et al., 2017; Yun, Vincelette, Acharya, & Abraham, 2017); (3) Patients who discontinued ibrutinib due to progression of disease may benefit from other small-molecule agents; however, those who progress with Richter’s transformation have an extremely poor prognosis, with an estimated life expectancy of 1.5 years (Mato et al., 2016). 

Pretreatment risk assessment is critical to mitigating risks and should include collaboration with co-managing providers such as PCPs and cardiologists to reduce potential drug-drug interactions and optimally manage expected adverse events. Factors shown to correlate with an increased risk of treatment-emergent atrial fibrillation include older age (≥ 75 years), male sex, valvular heart disease, and hypertension (Shanafelt et al., 2017; Yun et al., 2017). Anticoagulation, a proven method for reducing the potential for ischemic stroke in the setting of atrial fibrillation, can be safely managed in CLL patients receiving ibrutinib but requires vigilance and engagement of the patient in early reporting of adverse events and maintaining safety (Yun et al., 2017). Infections, particularly sinopulmonary infections, are common in the general CLL population. Atypical infections are also more common in CLL, owing to chronic immunosuppression associated with the disease and treatment. Vigilance in monitoring and methods to prevent atypical infections is recommended.

**Oral Adherence**

Based on the mechanism of action, the current recommendation for all small molecules, including ibrutinib, is to continue treatment until disease progression or unacceptable toxicity. Although scientifically sound, this principle presents many potential barriers to younger patients with a life expectancy of more than a couple of years, including adherence and unknown long-term toxicities (Barrientos, 2016). Oral adherence is a continuous challenge across cancer diagnoses. Patients missing ≥ 8 consecutive days of ibrutinib had a shorter median PFS than did those missing < 8 days (10.9 months vs. not reached; Barr et al., 2017).

Drug-drug interactions are common with small-molecule agents used to treat CLL and may either increase the risk of treatment-emergent adverse events, reduce the efficacy of novel agents, or modify the safety and efficacy of concomitant medications (Finnes et al., 2016).

A recent study evaluating perceptions of adherence communication and actual adherence in patients with CLL (81 physicians and 326 patients) showed a disparity between what physicians reported (87% indicated their CLL patients were "always" or "almost always" adherent) and patients reported (42% of patients reported they were "never" or "hardly ever" nonadherent to CLL treatment; Schnekel, Hallworth, Rider, Macomson, & McRae, 2017). 

The advanced practitioner in oncology plays a pivotal role in communication with the patient and among the interprofessional team. Establishing a program and process for support of patients on oral antineoplastic agents, including evaluation and management of possible drug-drug or drug-food interactions, is essential to safety and continued treatment with novel agents.

**Monoclonal Antibodies**

Monoclonal antibodies, including obinutuzumab (Gazyva), ofatumumab (Arzerra), and rituximab, provide a cadre of well-tolerated options for treatment of CLL in the front-line or RR setting (Chaoui et al., 2017; Wierda et al., 2017; Figure 2). They have become a mainstay in the treatment of lymphoid malignancies. Although they have limited benefit as single agents, when combined with standard chemotherapy (bendamustine, chlorambucil, fludarabine, cyclophosphamide), they provide safe and effective options for treatment with well-known and acceptable adverse events (Table 6). More recently, monoclonal antibodies are being combined with novel agents, with early data suggesting these "no chemotherapy required" regimens as feasible options for the treatment of CLL in the front-line, RR, and maintenance settings (Wierda et al., 2017; Table 5).

## TREATMENT OF RELAPSED OR RELAPSED/REFRACTORY CHRONIC LYMPHOCYTIC LEUKEMIA

Treatment of RRCLL requires re-evaluation of both patient- and disease-related factors. Analysis of prior therapies, response and tolerance of the prior therapies, any new or unresolved adverse events, and the presence of any acquired adverse disease features is essential. Let’s consider a patient with RRCLL.

**Case Study 4**

Mr. C is a 47-year-old male with a 10-year history of Rai stage I CLL with del(13q), ZAP-70–negative, normal β₂ microglobulin, and mutated IgHV at the time of diagnosis. He was previously treated with six cycles of FCR, achieving a complete response. It has been 7 years since his treatment. He presents to clinic with a new cervical lymph node, increased fatigue, abdominal pain, intermittent diarrhea, and shortness of breath when he bends over. His WBC count is 47 × 1,000/∝L with lymphocytosis, mild anemia (Hgb 11.2 g/dL), and a platelet count slightly below normal (137 × 1,000/∝L).

On physical exam, you note an enlarged spleen. Computed tomography scan of the chest, abdomen, and pelvis shows moderate splenomegaly and scattered adenopathy, with the abdomen with the largest nodal conglomerate in the mesentery (measuring 5.8 cm in greatest dimension). The FISH analysis shows the presence of del(17p). He has no other medical or surgical history. He is married, working full time, and has two children. He denies night sweats or unexplained weight loss.

 *Risk Assessment:* 

- CIRS: 0
- CLL-IPI score: 5
- The International CLL-IPI Working Group criteria for treatment: Yes
- Risk-adapted treatment selection: clinical trial, ibrutinib or venetoclax followed by allogeneic stem cell transplant

In patients who relapse more than 3 years after treatment with FCR or BR, repeating those regimens may be reasonable unless they have acquired del(17p) (Barrientos, 2016). The patient in case study 4 was treated more than 3 years ago with FCR and enjoyed a time-to-treatment interval of 7 years. However, he has an acquired del(17p), which changes his risk profile and the recommendation for treatment. Clonal evolution, the acquisition of new cytogenetic abnormalities during the disease course, is thought to drive CLL relapse and has implications for OS (Roberts & Huang, 2017; Woyach et al., 2014). The acquisition of high-risk abnormalities, particularly del(17p), is associated with inferior OS, whereas the acquisition of low- or intermediate-risk abnormalities (trisomy 12, deletion 13q) does not independently change the estimated OS. Therefore, all patients should be re-evaluated for acquired adverse-risk features and their level of fitness at the time of relapse or disease progression to effectively guide treatment decisions.

For this patient, a clinical trial with the endpoint being allogeneic stem cell transplant would be ideal. Alternatively, a regimen that incorporates agents with proven efficacy in patients with del(17p) CLL, either ibrutinib or venetoclax, as a bridge to transplant is optimal. Ibrutinib has been previously discussed in detail. Older or less fit patients, or those without del(17p), may be considered for alternative regimens (Figure 2). 

Idelalisib (Zydelig), a first-in-class, oral, selective, phosphoinositide 3-kinase (PI3K) δ inhibitor, was approved for the treatment of relapsed SLL in patients who have received at least two prior systemic therapies and for the treatment of relapsed CLL in combination with rituximab in patients for whom rituximab alone would be considered appropriate therapy because of other comorbidities (Gilead, 2016). Approval of idelalisib was based on a phase III randomized trial in a primarily older patient population (78% age > 65) with comorbidities (85% with CIRS > 6). Idelalisib offers a good option for selected patients in the second-line setting but must be balanced with a toxicity profile that includes a black box warning for fatal and severe hepatotoxicity, diarrhea or colitis, pneumonitis, and intestinal perforation (Gilead, 2016; Table 4). Ongoing trials are evaluating idelalisib in combination with monoclonal antibodies with and without novel agents (Table 5). Given the goal of transition to stem cell transplant and the risk of infections and gastrointestinal toxicity, this would not be the first choice of treatment in this young patient.

**Table 6:**
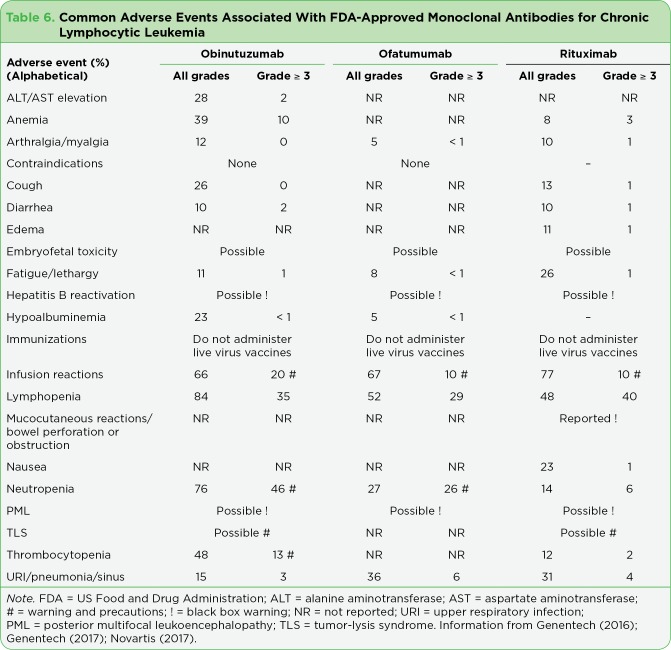
Common Adverse Events Associated With FDA-Approved Monoclonal Antibodies for Chronic Lymphocytic Leukemia

Venetoclax, a first-in-class small-molecule inhibitor of BCL2, is approved for the treatment of patients with CLL with 17p deletion, as detected by an FDA-approved test, who have received at least one prior therapy (AbbVie, 2016). Approval of venetoclax was based on data from phase I and II trials, with 79% of all patients in both trials achieving an objective response, regardless of the dose of venetoclax (Roberts & Huang, 2017). In addition, 20% of patients in these trials achieved a complete response, including patients with a del(17p) deletion. Although minimal residual disease (MRD) status was not included in the initial evaluation, a subset of patients achieved MRD-negative status.

Like other small-molecule agents, venetoclax has adverse events that require risk assessment and measures for prevention (Table 4). Tumor-lysis syndrome (TLS) is included in the warnings and precautions. Two fatal events and three cases of acute renal failure, with one requiring dialysis, were reported in the initial phase I trial. Subsequent modification of the dosing using a ramp-up method, together with guidelines for evaluation of TLS risk, prevention, and management, resulted in only 6% of patients on the phase II trial reported as having laboratory TLS. No cases of clinical TLS were noted (Roberts et al., 2016). The time to response with venetoclax is rapid, ranging from 0.1 to 8.1 months. Therefore, careful assessment of the risk of TLS prior to initiating therapy is required for safety. Patients at higher risk require inpatient administration of venetoclax and more intensive prevention and monitoring with the initial ramp-up. These guidelines can be found in the prescribing information for venetoclax and are also included in the National Comprehensive Cancer Network® Guidelines. As with other novel agents used in the treatment of CLL, venetoclax is being combined with other agents in ongoing clinical trials.

## CONCLUSIONS

Chronic lymphocytic leukemia is a heterogeneous disease with variability in prognosis, disease trajectory, and survival. This is primarily a disease of older adults. The robust pace of scientific development has improved our understanding of the underlying pathobiology of the disease and has elucidated actionable targets to produce novel therapies, ultimately expanding treatment options and in many cases improving therapeutic outcomes. None of these agents would be available in the absence of clinical trials. Therefore, every patient should be considered for a clinical trial regardless of the stage of disease or the line of therapy.

Treatment after failure of novel agents presents a challenge. Emerging trials using novel agents in rational combinations based on their mechanism of action, signaling pathways, and targets offer hope in generating therapies that more effectively exploit the vulnerabilities in CLL cells and their microenvironment. This will require lifelong learning on the part of all oncology professionals. 

Safe and effective integration of these new therapies presents a challenge for the advanced practitioner in oncology. Risk assessment and prognostication followed by risk-adapted treatment selection will promote selection of the best available therapy and limit overtreatment of patients who are unfit. The ability to anticipate, prevent, and identify adverse events, and then intervene promptly to mitigate risk, is critical to maximizing outcomes. Understanding the unique toxicity profiles of novel therapies and integrating this into risk-adapted treatment selection is critical to mitigating this risk. 

## SPECIAL NOTE: PRIMING THE PUMP

Condensing the plethora of scientific advances and implications for clinical practice into one article is impossible, and not all agents, regimens, or clinical trials have been reviewed. The Advanced Practitioner Society for Hematology and Oncology (APSHO) has embarked on a new educational and practice initiative entitled "Priming the Pump" (PTP). The PTP initiative is aimed at creating and maintaining an intuitive and interactive foundation of knowledge relative to emerging therapies. This initiative will include several comprehensive supplements to JADPRO, each focusing on a different disease state, covering discussion of the disease, relevant therapeutic pathways, targets, and treatment options, with the role of the advanced practitioner always at the core. 

The PTP supplement on CLL, to be published at the end of this year, will provide a comprehensive review of the pathobiology of CLL, diagnostic evaluation, risk-adapted treatment selection, management of adverse events, and discussion of ongoing clinical trials and emerging agents.

**Disclosure**

Ms. Kurtin has served as a consultant for AbbVie, Celgene, and Genentech. Dr. McBride has served on the speakers bureau for AbbVie. 

**How to Earn Credit**

To access the learning assessment and evaluation form online, visit http://meded.hbrsd.com/

**Statement of Credit:** Participants who successfully complete this activity (including scoring of a minimum of 70% on the learning assessment and complete and submit the evaluation form with an E-mail address) will be able to download a statement of credit.

## References

[1] AbbVie. Venclexta (venetoclax) package insert.. http://www.rxabbvie.com/pdf/venclexta.pdf.

[2] Balducci Lodovico, Dolan Dawn (2015). Chronic Lymphocytic Leukemia in the Elderly: Epidemiology and Proposed Patient-Related Approach.. *Cancer control : journal of the Moffitt Cancer Center*.

[3] Barr Paul M, Brown Jennifer R, Hillmen Peter, O’Brien Susan, Barrientos Jacqueline C, Reddy Nishitha M, Coutre Steven, Mulligan Stephen P, Jaeger Ulrich, Furman Richard R, Cymbalista Florence, Montillo Marco, Dearden Claire, Robak Tadeusz, Moreno Carol, Pagel John M, Burger Jan A, Suzuki Samuel, Sukbuntherng Juthamas, Cole George, James Danelle F, Byrd John C (2017). Impact of ibrutinib dose adherence on therapeutic efficacy in patients with previously treated CLL/SLL.. *Blood*.

[4] Barr P M, Robak T, Owen C J, Tedeschi A, Bairey O, Bartlett N L, Ghia P (2016). Updated efficacy and safety from the phase 3 Resonate-2 study: Ibrutinib as first line treatment option in patients 65 years and older with chronic lymphocytic leukemia/small lymphocytic lymphoma [Abstract 234].. *Blood (ASH Annual Meeting Abstracts)*.

[5] Barrientos Jacqueline C (2016). Sequencing of chronic lymphocytic leukemia therapies.. *Hematology. American Society of Hematology. Education Program*.

[6] Burger Jan A, Tedeschi Alessandra, Barr Paul M, Robak Tadeusz, Owen Carolyn, Ghia Paolo, Bairey Osnat, Hillmen Peter, Bartlett Nancy L, Li Jianyong, Simpson David, Grosicki Sebastian, Devereux Stephen, McCarthy Helen, Coutre Steven, Quach Hang, Gaidano Gianluca, Maslyak Zvenyslava, Stevens Don A, Janssens Ann, Offner Fritz, Mayer Jiří, O'Dwyer Michael, Hellmann Andrzej, Schuh Anna, Siddiqi Tanya, Polliack Aaron, Tam Constantine S, Suri Deepali, Cheng Mei, Clow Fong, Styles Lori, James Danelle F, Kipps Thomas J (2015). Ibrutinib as Initial Therapy for Patients with Chronic Lymphocytic Leukemia.. *The New England journal of medicine*.

[7] Burgler Simone (2015). Role of CD38 Expression in Diagnosis and Pathogenesis of Chronic Lymphocytic Leukemia and Its Potential as Therapeutic Target.. *Critical reviews in immunology*.

[8] Byrd J, Hillmen P, O’Brien S M, Barrientos J C, Reddy N M, Coutre S, Brown J R (2017). Long-term efficacy and safety with ibrutinib in previoulsy treated chronic lymphocytic leukemia: Up to four years of follow-up of the RESONATE study [Abstract 7510].. * Journal of Clinical Oncology (Meeting Abstracts)*.

[9] Byrd John C, Brown Jennifer R, O’Brien Susan, Barrientos Jacqueline C, Kay Neil E, Reddy Nishitha M, Coutre Steven, Tam Constantine S, Mulligan Stephen P, Jaeger Ulrich, Devereux Steve, Barr Paul M, Furman Richard R, Kipps Thomas J, Cymbalista Florence, Pocock Christopher, Thornton Patrick, Caligaris-Cappio Federico, Robak Tadeusz, Delgado Julio, Schuster Stephen J, Montillo Marco, Schuh Anna, de Vos Sven, Gill Devinder, Bloor Adrian, Dearden Claire, Moreno Carol, Jones Jeffrey J, Chu Alvina D, Fardis Maria, McGreivy Jesse, Clow Fong, James Danelle F, Hillmen Peter (2014). Ibrutinib versus ofatumumab in previously treated chronic lymphoid leukemia.. *The New England journal of medicine*.

[10] Byrd John C, Furman Richard R, Coutre Steven E, Flinn Ian W, Burger Jan A, Blum Kristie A, Grant Barbara, Sharman Jeff P, Coleman Morton, Wierda William G, Jones Jeffrey A, Zhao Weiqiang, Heerema Nyla A, Johnson Amy J, Sukbuntherng Juthamas, Chang Betty Y, Clow Fong, Hedrick Eric, Buggy Joseph J, James Danelle F, O’Brien Susan (2013). Targeting BTK with ibrutinib in relapsed chronic lymphocytic leukemia.. *The New England journal of medicine*.

[11] Chanan-Khan Asher, Cramer Paula, Demirkan Fatih, Fraser Graeme, Silva Rodrigo Santucci, Grosicki Sebastian, Pristupa Aleksander, Janssens Ann, Mayer Jiri, Bartlett Nancy L, Dilhuydy Marie-Sarah, Pylypenko Halyna, Loscertales Javier, Avigdor Abraham, Rule Simon, Villa Diego, Samoilova Olga, Panagiotidis Panagiots, Goy Andre, Mato Anthony, Pavlovsky Miguel A, Karlsson Claes, Mahler Michelle, Salman Mariya, Sun Steven, Phelps Charles, Balasubramanian Sriram, Howes Angela, Hallek Michael (2016). Ibrutinib combined with bendamustine and rituximab compared with placebo, bendamustine, and rituximab for previously treated chronic lymphocytic leukaemia or small lymphocytic lymphoma (HELIOS): a randomised, double-blind, phase 3 study.. *The Lancet. Oncology*.

[12] Chaoui Driss, Choquet Sylvain, Sanhes Laurence, Mahé Béatrice, Hacini Maya, Fitoussi Olivier, Arkam Yazid, Orfeuvre Hubert, Dilhuydy Marie-Sarah, Barry Marly, Jourdan Eric, Dreyfus Brigitte, Tempescul Adrian, Leprêtre Stéphane, Bardet Aurélie, Leconte Pierre, Maynadié Marc, Delmer Alain (2017). Relapsed chronic lymphocytic leukemia retreated with rituximab: interim results of the PERLE study.. *Leukemia & lymphoma*.

[13] Eichhorst Barbara, Fink Anna-Maria, Bahlo Jasmin, Busch Raymonde, Kovacs Gabor, Maurer Christian, Lange Elisabeth, Köppler Hubert, Kiehl Michael, Sökler Martin, Schlag Rudolf, Vehling-Kaiser Ursula, Köchling Georg, Plöger Christoph, Gregor Michael, Plesner Torben, Trneny Marek, Fischer Kirsten, Döhner Harmut, Kneba Michael, Wendtner Clemens-Martin, Klapper Wolfram, Kreuzer Karl-Anton, Stilgenbauer Stephan, Böttcher Sebastian, Hallek Michael (2016). First-line chemoimmunotherapy with bendamustine and rituximab versus fludarabine, cyclophosphamide, and rituximab in patients with advanced chronic lymphocytic leukaemia (CLL10): an international, open-label, randomised, phase 3, non-inferiority trial.. *The Lancet. Oncology*.

[14] Eichhorst Barbara, Hallek Michael (2016). Prognostication of chronic lymphocytic leukemia in the era of new agents.. *Hematology. American Society of Hematology. Education Program*.

[15] Farooqui Mohammed Z H, Valdez Janet, Martyr Sabrina, Aue Georg, Saba Nakhle, Niemann Carsten U, Herman Sarah E M, Tian Xin, Marti Gerald, Soto Susan, Hughes Thomas E, Jones Jade, Lipsky Andrew, Pittaluga Stefania, Stetler-Stevenson Maryalice, Yuan Constance, Lee Yuh Shan, Pedersen Lone B, Geisler Christian H, Calvo Katherine R, Arthur Diane C, Maric Irina, Childs Richard, Young Neal S, Wiestner Adrian (2015). Ibrutinib for previously untreated and relapsed or refractory chronic lymphocytic leukaemia with TP53 aberrations: a phase 2, single-arm trial.. *The Lancet. Oncology*.

[16] Finnes Heidi D, Chaffee Kari G, Call Timothy G, Ding Wei, Kenderian Saad S, Bowen Deborah A, Conte Michael, McCullough Kristen B, Merten Julianna A, Bartoo Gabriel T, Smith Matthew D, Leis Jose, Chanan-Khan Asher, Schwager Susan M, Slager Susan L, Kay Neil E, Shanafelt Tait D, Parikh Sameer A (2017). Pharmacovigilance during ibrutinib therapy for chronic lymphocytic leukemia (CLL)/small lymphocytic lymphoma (SLL) in routine clinical practice.. *Leukemia & lymphoma*.

[17] Foluso Ogunleye, Glick Alexander, Stender Michael, Jaiyesimi Ishmael (2016). Ibrutinib as a Bruton Kinase Inhibitor in the Management of Chronic Lymphocytic Leukemia: A New Agent With Great Promise.. *Clinical lymphoma, myeloma & leukemia*.

[18] Fischer K, Cramer P, Busch R, Bottcher S, Bahlo J, Schubert J, Wendtner C M (2012). Bendamustine in combination with rituximab for previously untreated patients with chronic lymphocytic leukemia: A multicenter phase II trial of the German Chronic Lymphocytic Leukemia Study Group.. *Journal of Clinical Oncology*.

[19] Gashonia L M, Carver J R, O’Quinn R, Clasen S, Hughes M E, Schuster S J, Mato A R (2017). Persistence of ibrutinib-associated hypertension in CLL pts treated in a real-world experience [Abstract 7525].. *Journal of Clinical Oncology (Meeting Abstracts)*.

[20] Genentech. (2016). Gazyva (obinutuzumab) package insert.. http://www.gene.com/download/pdf/gazyva_prescribing.pdf.

[21] Genentech. (2017). Genentech.. http://www.gene.com.

[22] Gilead. (2016). Zydelig (idelalisib) package insert.. http://www.gilead.com/~/media/Files/pdfs/medicines/oncology/zydelig/zydelig_pi.pdf.

[23] Gilead. (2017). Gilead Sciences, Inc.. http://www.gilead.com/.

[24] Goede Valentin, Cramer Paula, Busch Raymonde, Bergmann Manuela, Stauch Martina, Hopfinger Georg, Stilgenbauer Stephan, Döhner Hartmut, Westermann Anne, Wendtner Clemens M, Eichhorst Barbara, Hallek Michael (2014). Interactions between comorbidity and treatment of chronic lymphocytic leukemia: results of German Chronic Lymphocytic Leukemia Study Group trials.. *Haematologica*.

[25] Gruber Michaela, Wu Catherine J (2014). Evolving understanding of the CLL genome.. *Seminars in hematology*.

[26] Guièze Romain, Wu Catherine J (2015). Genomic and epigenomic heterogeneity in chronic lymphocytic leukemia.. *Blood*.

[27] Hallek Michael (2015). Chronic lymphocytic leukemia: 2015 Update on diagnosis, risk stratification, and treatment.. *American journal of hematology*.

[28] The International CLL-IPI Working Group. (2016). An international prognostic index for patients with chronic lymphocytic leukaemia (CLL-IPI): a meta-analysis of individual patient data.. *The Lancet. Oncology*.

[29] Jain Preetesh, Thompson Philip A, Keating Michael, Estrov Zeev, Ferrajoli Alessandra, Jain Nitin, Kantarjian Hagop, Burger Jan A, O’Brien Susan, Wierda William G (2017). Long-term outcomes for patients with chronic lymphocytic leukemia who discontinue ibrutinib.. *Cancer*.

[30] Kunk P R, Mock J, Devitt M E, Palkimas S, Sen J, Portell C A, Williams M E (2016). Major bleeding with ibrutinib more than expected? [Abstract 3229].. *Blood (ASH Annual Meeting Abstracts)*.

[31] Maddocks Kami, Jones Jeffrey A (2016). Bruton tyrosine kinase inhibition in chronic lymphocytic leukemia.. *Seminars in oncology*.

[32] Mato A R, Lamanna N, Ujjani C S, Brander D M, Hill B T, Howlett C, Barr P (2016). Toxicities and outcomes of ibrutinib-treated patients in the United States: Large retrospective analysis of 621 real world patients [Abstract 3222].. *Blood (ASH Annual Meeting Abstracts)*.

[33] Miller M D, Paradis C F, Houck P R, Mazumdar S, Stack J A, Rifai A H, Mulsant B, Reynolds C F (1992). Rating chronic medical illness burden in geropsychiatric practice and research: application of the Cumulative Illness Rating Scale.. *Psychiatry research*.

[34] Montserrat Emili, Bauman Tycho, Delgado Julio (2016). Present and future of personalized medicine in CLL.. *Best practice & research. Clinical haematology*.

[35] Novartis. (2017). Novartis pharmaceuticals.. https://www.hcp.novartis.com/.

[36] O’Brien Susan, Furman Richard R, Coutre Steven E, Sharman Jeff P, Burger Jan A, Blum Kristie A, Grant Barbara, Richards Donald A, Coleman Morton, Wierda William G, Jones Jeffrey A, Zhao Weiqiang, Heerema Nyla A, Johnson Amy J, Izumi Raquel, Hamdy Ahmed, Chang Betty Y, Graef Thorsten, Clow Fong, Buggy Joseph J, James Danelle F, Byrd John C (2014). Ibrutinib as initial therapy for elderly patients with chronic lymphocytic leukaemia or small lymphocytic lymphoma: an open-label, multicentre, phase 1b/2 trial.. *The Lancet. Oncology*.

[37] Parikh Sameer A, Shanafelt Tait D (2014). Risk factors for Richter syndrome in chronic lymphocytic leukemia.. *Current hematologic malignancy reports*.

[38] Parikh Sameer A, Shanafelt Tait D (2016). Prognostic factors and risk stratification in chronic lymphocytic leukemia.. *Seminars in oncology*.

[39] Parikh Sameer A, Strati Paolo, Tsang Mazie, West Colin P, Shanafelt Tait D (2016). Should IGHV status and FISH testing be performed in all CLL patients at diagnosis? A systematic review and meta-analysis.. *Blood*.

[40] Pflug Natali, Bahlo Jasmin, Shanafelt Tait D, Eichhorst Barbara F, Bergmann Manuela A, Elter Thomas, Bauer Kathrin, Malchau Gebhart, Rabe Kari G, Stilgenbauer Stephan, Döhner Hartmut, Jäger Ulrich, Eckart Michael J, Hopfinger Georg, Busch Raymonde, Fink Anna-Maria, Wendtner Clemens-Martin, Fischer Kirsten, Kay Neil E, Hallek Michael (2014). Development of a comprehensive prognostic index for patients with chronic lymphocytic leukemia.. *Blood*.

[41] Pharmacyclics. (2016). Imbruvica (ibrutinib) package insert.. https://www.imbruvica.com/prescribing-information.

[42] Rai Kanti R, Jain Preetesh (2016). Chronic lymphocytic leukemia (CLL)-Then and now.. *American journal of hematology*.

[43] Rai K R, Sawitsky A, Cronkite E P, Chanana A D, Levy R N, Pasternack B S (1975). Clinical staging of chronic lymphocytic leukemia.. *Blood*.

[44] Roberts Andrew W, Davids Matthew S, Pagel John M, Kahl Brad S, Puvvada Soham D, Gerecitano John F, Kipps Thomas J, Anderson Mary Ann, Brown Jennifer R, Gressick Lori, Wong Shekman, Dunbar Martin, Zhu Ming, Desai Monali B, Cerri Elisa, Heitner Enschede Sari, Humerickhouse Rod A, Wierda William G, Seymour John F (2016). Targeting BCL2 with Venetoclax in Relapsed Chronic Lymphocytic Leukemia.. *The New England journal of medicine*.

[45] Roberts A W, Huang Dcs (2017). Targeting BCL2 With BH3 Mimetics: Basic Science and Clinical Application of Venetoclax in Chronic Lymphocytic Leukemia and Related B Cell Malignancies.. *Clinical pharmacology and therapeutics*.

[46] Roos-Weil Damien, Nguyen-Khac Florence, Bernard Olivier A (2016). Chronic lymphocytic leukemia: Time to go past genomics?. *American journal of hematology*.

[47] Rossi Davide, Gerber Bernhard, Stüssi Georg (2017). Predictive and prognostic biomarkers in the era of new targeted therapies for chronic lymphocytic leukemia.. *Leukemia & lymphoma*.

[48] Sarfati Diana, Koczwara Bogda, Jackson Christopher (2016). The impact of comorbidity on cancer and its treatment.. *CA: a cancer journal for clinicians*.

[49] Schnekel B, Hallworth P, Rider A, Macomson B, McRae J (2017). Comparing adherence levels of CLL treatment from both the patient and physician perspective in the U.S. [Abstract e19009]..

[50] Shanafelt Tait D, Parikh Sameer A, Noseworthy Peter A, Goede Valentin, Chaffee Kari G, Bahlo Jasmin, Call Timothy G, Schwager Susan M, Ding Wei, Eichhorst Barbara, Fischer Kirsten, Leis Jose F, Chanan-Khan Asher Alban, Hallek Michael, Slager Susan L, Kay Neil E (2017). Atrial fibrillation in patients with chronic lymphocytic leukemia (CLL).. *Leukemia & lymphoma*.

[51] Siegel Rebecca L, Miller Kimberly D, Jemal Ahmedin (2017). Cancer Statistics, 2017.. *CA: a cancer journal for clinicians*.

[52] Stauder R, Eichhorst B, Hamaker M E, Kaplanov K, Morrison V A, Österborg A, Poddubnaya I, Woyach J A, Shanafelt T, Smolej L, Ysebaert L, Goede V (2017). Management of chronic lymphocytic leukemia (CLL) in the elderly: a position paper from an international Society of Geriatric Oncology (SIOG) Task Force.. *Annals of oncology : official journal of the European Society for Medical Oncology*.

[53] Thompson Philip A, Tam Constantine S, O’Brien Susan M, Wierda William G, Stingo Francesco, Plunkett William, Smith Susan C, Kantarjian Hagop M, Freireich Emil J, Keating Michael J (2016). Fludarabine, cyclophosphamide, and rituximab treatment achieves long-term disease-free survival in IGHV-mutated chronic lymphocytic leukemia.. *Blood*.

[54] Wierda William G (2015). Updates to the management of chronic lymphocytic leukemia.. *Journal of the National Comprehensive Cancer Network : JNCCN*.

[55] Wierda William G, Zelenetz Andrew D, Gordon Leo I, Abramson Jeremy S, Advani Ranjana H, Andreadis C Babis, Bartlett Nancy, Byrd John C, Caimi Paolo, Fayad Luis E, Fisher Richard I, Glenn Martha J, Habermann Thomas M, Harris Nancy Lee, Hernandez-Ilizaliturri Francisco, Hoppe Richard T, Horwitz Steven M, Kaminski Mark S, Kelsey Christopher R, Kim Youn H, Krivacic Susan, LaCasce Ann S, Martin Michael G, Nademanee Auayporn, Porcu Pierluigi, Press Oliver, Rabinovitch Rachel, Reddy Nishitha, Reid Erin, Roberts Kenneth, Saad Ayman A, Snyder Erin D, Sokol Lubomir, Swinnen Lode J, Vose Julie M, Yahalom Joachim, Dwyer Mary A, Sundar Hema (2017). NCCN Guidelines Insights: Chronic Lymphocytic Leukemia/Small Lymphocytic Leukemia, Version 1.2017.. *Journal of the National Comprehensive Cancer Network : JNCCN*.

[56] Woyach Jennifer A, Johnson Amy J (2015). Targeted therapies in CLL: mechanisms of resistance and strategies for management.. *Blood*.

[57] Yun S, Vincelette N D, Acharya U, Abraham I (2017). Risk of atrial fibrillation and bleeding diathesis associated with ibrutinib treatment: A systematic review and pooled analysis of four randomized controlled trials.. *Clinical Lymphoma, Myeloma & Leukemia*.

